# 5‐Hydroxymethylfurfural reduces skeletal muscle superoxide production and modifies force production in rats exposed to hypobaric hypoxia

**DOI:** 10.14814/phy2.15743

**Published:** 2023-07-25

**Authors:** Geoffrey E. Ciarlone, Joshua M. Swift, Brian T. Williams, Richard T. Mahon, Nicholas G. Roney, Tianzheng Yu, Heath G. Gasier

**Affiliations:** ^1^ Undersea Medicine Department Naval Medical Research Center Silver Spring Maryland USA; ^2^ Department of Military & Emergency Medicine Uniformed Services University of the Health Sciences Bethesda Maryland USA; ^3^ The Henry M. Jackson Foundation for the Advancement of Military Medicine, Inc Bethesda Maryland USA; ^4^ The Duke Center for Hyperbaric Medicine & Environmental Physiology Duke University Durham North Carolina USA

**Keywords:** antioxidants, high altitude, mitochondria, oxidant stress

## Abstract

Decreased blood‐tissue oxygenation at high altitude (HA) increases mitochondrial oxidant production and reduces exercise capacity. 5‐Hydroxymethylfurfural (5‐HMF) is an antioxidant that increases hemoglobin's binding affinity for oxygen. For these reasons, we hypothesized that 5‐HMF would improve muscle performance in rats exposed to a simulated HA of ~5500 m. A secondary objective was to measure mitochondrial activity and dynamic regulation of fission and fusion because they are linked processes impacted by HA. Fisher 344 rats received 5‐HMF (40 mg/kg/day) or vehicle during exposure to sea level or HA for 72 h. Right ankle plantarflexor muscle function was measured pre‐ and post‐exposure. Post‐exposure measurements included arterial blood gas and complete blood count, flexor digitorum brevis myofiber superoxide production and mitochondrial membrane potential (ΔΨm), and mitochondrial dynamic regulation in the soleus muscle. HA reduced blood oxygenation, increased superoxide levels and lowered ΔΨm, responses that were accompanied by decreased peak isometric torque and force production at frequencies >75 Hz. 5‐HMF increased isometric force production and lowered oxidant production at sea level. In HA exposed animals, 5‐HMF prevented a decline in isometric force production at 75–125 Hz, prevented an increase in superoxide levels, further decreased ΔΨm, and increased mitochondrial fusion 2 protein expression. These results suggest that 5‐HMF may prevent a decrease in hypoxic force production during submaximal isometric contractions by an antioxidant mechanism.

## Graphical abstract

High altitude (HA) exposure affects all phases in oxygen transport that increases mitochondrial oxidant production and negatively impacts exercise performance. 5‐Hydroxymethylfurfural (5‐HMF) is a natural compound that increases blood oxygenation and functions as an antioxidant. Here, 5‐HMF reduced skeletal muscle oxidant production and prevented a decline in isometric force production at 75–125 Hz after a 72 h exposure to a simulated HA of 5500 m.

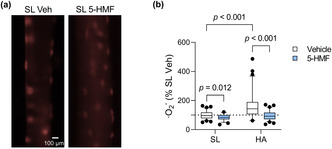

## 1 INTRODUCTION

The atmospheric partial pressure of oxygen (po_2_) is reduced during ascent to high altitude (HA, >2438 m) due to a fall in barometric pressure, causing a decrease in po_2_ at each point along the oxygen transport cascade (Calbet & Lundby, 2009; Richardson et al., 2006). The ensuing hypoxia causes a reduction in peak power output and maximal voluntary isometric contraction torque during and after high intensity repeated sprint exercise, respectively (Balsom et al., 1994; Bowtell et al., 2014; Girard et al., 2016; Goods et al., 2014). These decrements are accompanied by reduced oxygen consumption (VO_2_) during sprints and rest intervals, potentially resulting in a mismatch between ATP demand and synthesis (Balsom et al., 1994; Bowtell et al., 2014; Hogan et al., 1999). This may occur because of increased phosphocreatine hydrolysis and reduced oxidative phosphorylation, placing a greater reliance on rapid (non‐oxidative) glycolysis (Hogan et al., 1999; Magalhães et al., 2005; Parolin et al., 2000). However, the capacity to regenerate ATP exceeds consumption during sprint cycling performed for 10 s after incremental cycling in hypoxia and bilateral leg occlusion, questioning a bioenergetic limitation for at least sprints of very short duration (Morales‐Alamo et al., 2015).

Another potential cause of muscle fatigue in hypoxia is increased oxidant production (Reid, 2016). Most of the VO_2_ serves as the terminal electron acceptor from cytochrome c oxidase for oxidative phosphorylation, while some oxygen undergoes one‐electron reduction producing superoxide anions (Murphy, 2009). Formation of superoxide is proportional to oxygen concentration, thus a decrease in po_2_ should lower superoxide levels. In hypoxia, however, decreased oxygen availability leads to augmented superoxide production (Chandel et al., 2000; Guzy et al., 2005; Magalhães et al., 2005). The increase is accompanied by reduced skeletal muscle mitochondrial membrane potential (ΔΨm) and respiratory capacity, a shift in mitochondrial dynamic regulation towards fission, and decreased mitochondrial DNA content and biogenesis (Magalhães et al., 2005; Zou et al., 2014, 2015). Increased mitochondrial oxidant production also impacts force production by decreasing myofibrillar calcium sensitivity (Gandra et al., 2018). Preserving skeletal muscle po_2_ or antioxidant activity during HA should improve redox balance and lower decrements in muscle performance.

5‐hydroxymethylfurfural (5‐HMF) is a six carbon compound formed from reducing sugars in honey and processed foods (Shapla et al., 2018). 5‐HMF binds to the N‐terminal valine of hemoglobin with high affinity, allosterically shifting the oxygen hemoglobin dissociation curve to the left (Abdulmalik et al., 2005; Mahon et al., 2019; Woyke et al., 2021). In normoxia, lowering the P_50_ does not favor oxygen release. A benefit may exist in hypoxia since a higher arterial blood oxygen saturation will increase diffusion gradients and tissue po_2_ (Li et al., 2011; Yalcin & Cabrales, 2012). Moreover, the reactive groups within 5‐HMF's furan ring attract electrons, making it an antioxidant (Li et al., 2011; Shapla et al., 2018). While 5‐HMF and its byproduct 5‐sulfo‐oxymethylfurfural are carcinogens and cytotoxic, levels in the range of 80–100 mg/kg body weight are considered safe (Abraham et al., 2011; Shapla et al., 2018). From this information, we hypothesized that 5‐HMF would reduce skeletal muscle superoxide production and improve hindlimb muscle performance after exposure to a simulated HA of 5500 m for 72 h. A secondary objective was to measure skeletal muscle mitochondrial activity and dynamic regulation of fission and fusion.

## 2 MATERIALS AND METHODS

### 2.1 Experimental design

**FIGURE 1:**
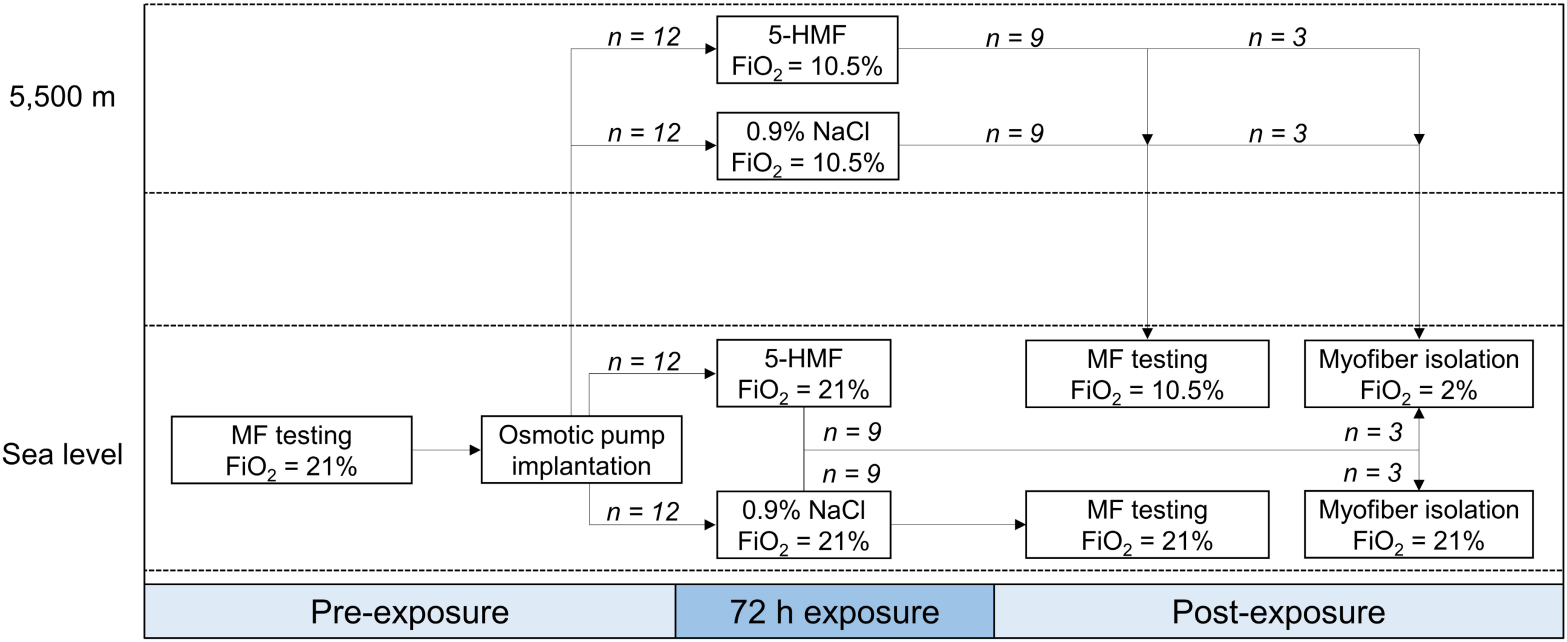
Experimental design. MF, muscle function.

Forty‐eight male Fisher 344 rats (Charles River Laboratories) between the ages of 10–12 weeks and weighing 200–225 g remained on a 12:12 light:dark cycle with free access to food and water. The experimental design is shown in Figure 1. Rats (*n* = 36) were anesthetized and underwent plantarflexor muscle function testing. The next morning rats were anesthetized for implantation of osmotic pumps containing 0.9% NaCl or 5‐HMF (40 mg/kg/day). Within 30 min of recovering from anesthesia, animals were placed in chambers and exposed to sea level or a simulated HA of ~5500 m for 72 h. This level of hypobaric hypoxia was selected because it increases pulmonary arterial pressure and causes hypophagia in rats, resembling the effects of HA in humans (Ou et al., 1986; Schnakenberg & Rogers, 1982). Every 24 h rats were placed in different chambers containing the same fraction of inspired oxygen (FiO_2_) for cleaning, weighing of food, and replenishment of food and water. After 72 h rats were anesthetized and muscle function testing was repeated. Blood was collected via cardiac puncture for measurement of complete blood count and arterial blood gas. In the contralateral limb, the gastrocnemius, plantaris, and soleus muscles were collected, weighed, and frozen. An additional group of rats (*n* = 12) completed air and HA exposures only, and were used for live cell imaging experiments. For molecular analysis, the soleus muscle was selected because it contains mostly slow myosin heavy chain (MHC) (Type I, 97%) and is rich in mitochondria, thus highly susceptible to oxidant damage (Bloemberg & Quadrilatero, 2012). Myofibers isolated from the flexor digitorum brevis (FDB) muscle, which is also a plantarflexor, were used for measurement of superoxide and ΔΨm with fluorescence microscopy because of their high viability and MHC composition that resembles the gastrocnemius and plantaris complex (Bloemberg & Quadrilatero, 2012; Komiya et al., 2015). All rats were euthanized by isoflurane and cardiac puncture.

“The study protocol was reviewed and approved by the Walter Reed Army Institute of Research/Naval Medical Research Center Institutional Animal Care and Use Committee in compliance with all Federal regulations governing the protection of animals and research. The health status of animals was monitored daily, and the research was conducted in a facility accredited by the Association for Assessment and Accreditation of Laboratory Animal Care‐International. Euthanasia was carried out in accordance with the recommendations and guidelines of the American Veterinary Medical Association.”

### 2.2 In‐situ muscle function testing

Right ankle plantarflexor muscle function was measured using a rodent isokinetic dynamometer as previously described (Swift et al., 2010). Before and following exposures, rats were anesthetized with 2% isoflurane and the right hindlimb was shaved and cleaned. Two monopolar needle EMG electrodes (Chalgren 125–425‐36TP) were inserted distal to the hip along the sciatic nerve. The right foot was secured to a force plate connected to an isometric dynamometer for recording of plantarflexion peak torque, force‐frequency and fatigability (Aurora Scientific). The knee was secured vertically by a clamp to standardize hind‐limb placement relative to the force plate. The following hardware and software (Aurora Scientific, Inc.) were used to determine force output: a High‐Power, Bi‐Phase Stimulator (Model No. 701C) and Dual‐Mode Lever System (Model No. 305C‐LR‐FP), 610A Dynamic Muscle Control software (v5.300), 611A Dynamic Muscle Analysis software (v5.100), and 612A DMA‐High Throughput Module. Peak torque was measured using 175 Hz. Current to the electrode was tested over multiple values to determine optimal current input (generally 5–6 mA). Each maximal contraction was separated by a 2 min rest period. Following a 2 min rest interval, force‐frequency curves were developed by measuring force output at 10, 20, 30, 40, 50, 70, 100, 125, 150, and 200 Hz (30 s rest intervals between frequencies). After another 2 min rest interval, 30 maximal contractions at 175 Hz (5 s rest interval) were performed to determine muscle fatigue. Immediately after air and HA exposures, force output testing was repeated similarly, except that HA rats breathed a hypoxic gas mixture (10.5% O_2_, 0.04% CO_2_, balance N_2_) to match the FiO_2_ at 5500 m. This was achieved by mixing air with pure N_2_ and confirmed by a gas analyzer. Total time for testing was 15–20 min per animal.

### 2.3 Treatment delivery

5‐HMF (Millipore Sigma, #W501808) or 0.9% NaCl (vehicle) were transferred to osmotic pumps (Alzet Model 2001) set to deliver 40 mg/kg body weight per day and placed in warm saline overnight for priming per manufacturer's instructions. The following day rats were anesthetized a second time with 2% isoflurane, and the area between the scapula was shaved and the skin was cleaned. Bupivacaine hydrochloride (4 mg/kg) was injected into the site and a small lateral incision was made along the center of the back. The pump was inserted into the subcutaneous pocket and the incision site was closed with staples. Buprenorphine (0.02–0.5 mg/kg) was administered subcutaneously for prophylactic analgesia and the animals recovered from anesthesia within 30 min.

### 2.4 Sea level and HA exposures

Rats were placed individually in cylindrical Plexiglas chambers and decompressed to ~5500 m (300 m/min) or remained at sea level pressure for 72 h (26°C). Every 24 h the chambers were cleaned, the rat's food and body weight were recorded, and the food and water replenished (~40 min). HA rats were compressed to sea level at a rate of 300 m/min. Rats were placed in temporary chambers and breathed the same normoxic or hypoxic gas mixtures during this time.

### 2.5 Blood and tissue collection

Following exposures and muscle function testing, rats were removed from the force plate and transferred to a surgical table breathing the same normoxic or hypoxic gas mixtures with isoflurane (Abraham et al., 2011). Blood was collected into heparinized 1 mL syringes via cardiac puncture for measurement of complete blood count (Sysmex XT‐2000*i*) and arterial blood gas (ABL90 Flex Plus Radiometer). The plantarflexors (gastrocnemius, plantaris and soleus) were harvested, weighed, and flash frozen in liquid N_2_ and stored at −80°C. Plantarflexor muscle weights were used to measure the degree of muscle atrophy.

### 2.6 Superoxide levels and mitochondrial membrane potential

Following exposures, FDB muscles were collected from rats in each group and placed into 2 mL vials containing deoxygenated (2% O_2_) phosphate buffer saline. The po_2_ was estimated to match intramuscular po_2_ at ~5500 m. The samples were transported to the Uniformed Services University of the Health Sciences within 60 min of collection for isolation of myofibers as previously described (Dohl et al., 2018; Yu et al., 2019). HA FDB muscles were incubated in a hypoxic chamber (2% O_2_, 5% CO_2_, balanced N_2_) placed within the cell culture incubator maintained at 37°C. Superoxide was measured using 5 μM dihydroethidium (DHE) (Thermo Fisher Scientific, Cat. #D23107) (Ex: 500/Em: 588). Mitochondrial membrane potential was measured using 100 nM of tetramethylrhodamine ethyl ester (TMRE) (Thermo Fisher Scientific, Cat. #T669) (Ex: 555/Em: 579) (Brand & Nicholls, 2011). On experimental days, ~20–30 healthy myofibers were imaged using a Nikon Ti‐E epifluorescence microscope (Nikon) and sCMOS pco.edge 4.2 digital camera (PCO AG). Experiments were repeated on three separate occasions. Fluorescence intensity was quantified using ImageJ software (NIH).

### 2.7 Immunoblotting

Polyacrylamide gel electrophoresis was performed with 10 μg of protein from soleus muscle. The following primary antibodies (1:1000) were used: mouse anti‐total OXPHOS (RRID:AB_2629281, Abcam), rabbit anti‐dynamin‐related protein 1 (DRP1, RRID:AB_10950498, Cell Signaling), rabbit anti‐optic atrophy 1 (OPA1, RRID:AB_2799728, Cell Signaling), rabbit anti‐mitofusion 1 (MFN1, RRID:AB_2250540, Santa Cruz Biotechnology), rabbit anti‐mitofusion 2 (MFN2, RRID:AB_2142754, Santa Cruz Biotechnology) and rabbit anti‐GAPDH (RRID:AB_10806772, Millipore). The following secondary antibodies were used (1:10,000): goat anti‐mouse (RRID:AB_2617113, Bio‐Rad) and goat anti‐rabbit (RRID:AB_11125142, Bio‐Rad). Membranes were imaged with a Bio‐Rad ChemiDoc MP Imaging System. Densitometry quantification was performed using ImageJ software.

### 2.8 RT‐qPCR

RNA was isolated from ~25 mg of soleus muscle using TRIzol® Reagent (ThermoFisher Scientific). One microgram of RNA was used for first strand cDNA synthesis using QuantiTect Reverse Transcription Kits (Qiagen, Cat# 205311) and amplified in a TaqMan PCR Master Mix (Cat# 201445) with the following Thermo Fisher Scientific probes and primers: *Opa1* (Cat# 4331182), *Mfn1* (Cat# 4331182), *Mfn2* (Cat# 4331182), dynamin‐related protein 1 (*Dnm1l*, Cat# 4331182), mitochondrial fission 1 (*Fis1*, Cat# 4331182) and GAPDH (Cat# 4351368). qPCR was completed using an iCyclerQ (Bio‐Rad). Gene cycle threshold (Ct) values were normalized to GAPDH and data were quantified by the 2^−ΔΔ*CT*^ method.

### 2.9 Statistical analysis

Statistical power and sample size were determined from mean peak isometric torque measurements obtained in our unpublished preliminary experiments. From these data it was determined that 9–10 rats/group were required to detect an approximate difference of 20% with an alpha of 0.05 and 80% power. Body weight, food intake, and muscle stimulation data were analyzed using a two‐factor repeated measures ANOVA. All other data were analyzed using a two‐factor ANOVA. A Bonferroni *t*‐test was used for pairwise multiple comparisons. Data were log transformed if normality and variance homogeneity assumptions were not met. Data are presented as medians with quartiles, individual data points with medians and quartiles, and means ± SD or SE. Values of *p* < 0.05 were considered statistically significant. Data were analyzed using SigmaPlot version 14.0 (Systat Software).

## 3 RESULTS

### 3.1 Food consumption, body weight, and muscle wet weights

**FIGURE 2:**
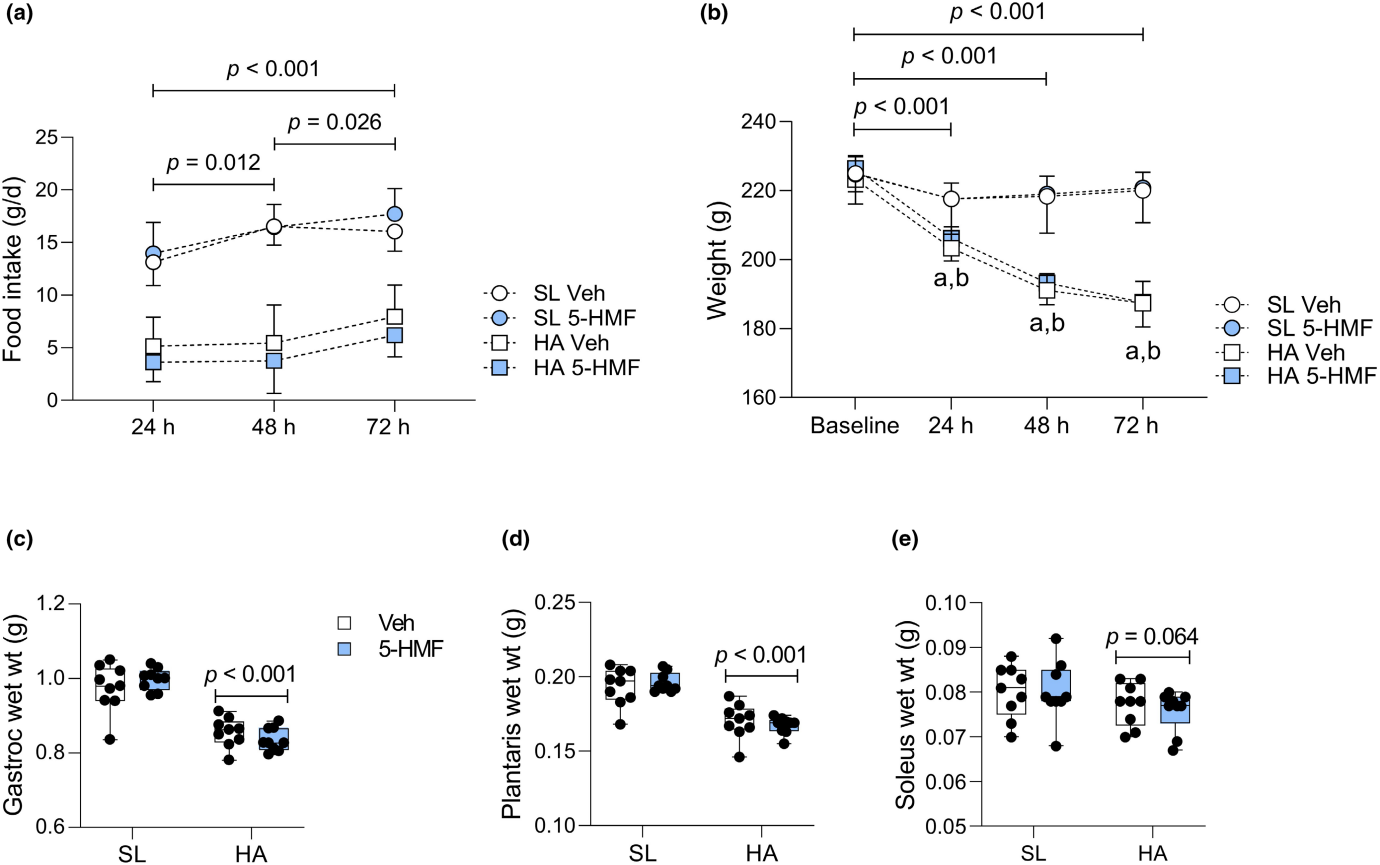
Food intake, body weight and muscle wet weights recorded before, during and after normobaric normoxic and hypobaric hypoxic exposures. (a) Food consumption and (b) body weight. Values are means ± SD, *n* = 9/group. Main effect of HA and time for food intake, *p* < 0.001. Main effect of HA, time, and HA × time for body weight, *p* < 0.001. ^a^Between HA Veh and sea level (SL) Veh; ^b^between HA 5‐HMF and SL 5‐HMF. (c) Gastroc (gastrocnemius), (d) plantaris and (e) soleus wet weights. Values are individual data points with interquartile ranges (whiskers represent min to max values), *n* = 9/group. Main effect of HA for gastrocnemius and plantaris muscles (shown).

Exposing rats to a simulated HA of ~5500 m for 72 h led to a 58%–72% reduction in food intake (Figure 2a), a 9%–16% reduction in body weight (Figure 2b), and atrophy of the gastrocnemius (−14%) and plantaris (−13%) muscles independent of treatment (Figure 2c,d). There was a trend for a reduction (~5%) in soleus muscle weight in HA rats (Figure 2e).

### 3.2 Arterial blood gas and complete blood count

**FIGURE 3:**
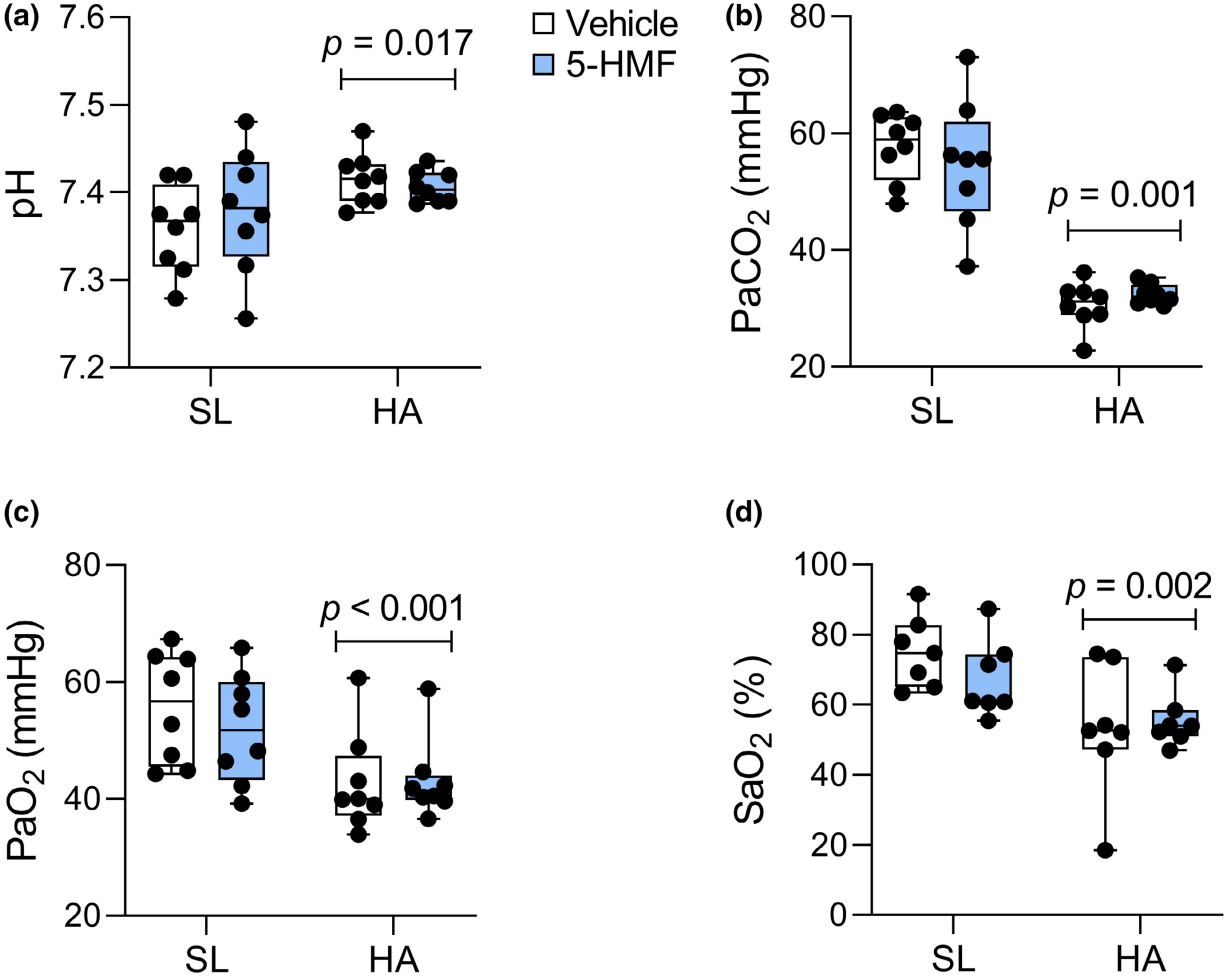
Arterial blood gas measured in anesthetized rats breathing normobaric normoxic or hypoxic gas. (a) pH, (b) PaCO_2_, (c) PaO_2_, and (d) SaO_2_. Values are individual data points with interquartile ranges (whiskers represent min to max values), *n* = 7‐8/group. Main effect of HA (shown).

Arterial blood gas values were within the expected range when isoflurane anesthesia is delivered in 21% oxygen at sea level (Wilding et al., 2017). In HA exposed rats breathing a FiO_2_ of 10.5%, arterial blood pH increased by 0.042 units and PaO_2_, SaO_2_, and PaCO_2_ fell by 20%, 23%, and 44%, respectively (Figure 3). These values are consistent with those reported by others (Jacobson & Dallman, 1989). 5‐HMF did not alter ABG responses to HA. Analysis of complete blood count showed an effect of HA on leukocytes (+37%), erythrocytes (+9%), hemoglobin (+11%), hematocrit (+15%), platelets (+41%) and neutrophils (+77%), and lymphocytes (−18%) (Table 1). These changes likely reflect dehydration and hemoconcentration. 5‐HMF increased the concentration of monocytes by 24%.

**TABLE 1:** Complete blood count.

Parameter	SL Veh	SL 5‐HMF	HA Veh	HA 5‐HMF
WBC (10^3^/μL)	6.0 ± 1.0	5.6 ± 1.0	8.2 ± 1.2*	7.7 ± 2.7*
RBC (10^6^/μL)	9.0 ± 0.4	8.7 ± 0.3	9.3 ± 1.8*	9.9 ± 0.5*
Hb (g/dL)	15.7 ± 0.7	15.1 ± 0.6	16.5 ± 3.0**	17.5 ± 1.1**
HCT (%)	45.8 ± 3.5	43.6 ± 1.3	50.1 ± 8.2***	52.6 ± 2.4***
PLT (10^3^/μL)	803 ± 70	724 ± 135	1086 ± 84***	1070 ± 297***
Neut (%)	19.5 ± 3.8	18.6 ± 2.4	32.6 ± 3.5***	34.9 ± 5.8***
Lymph (%)	75.4 ± 3.7	75.6 ± 1.6	63.6 ± 3.2***	60.0 ± 6.8***
Mono (%)	3.4 ± 0.8	4.0 ± 1.6^#^	2.8 ± 0.5	3.7 ± 0.8^#^

*Note*: Blood was collected via cardiac puncture following muscle performance testing. Values are means ± SD. Main effect of high altitude (HA): *p < 0.05, **p < 0.01, ***p < 0.001. Main effect of 5‐HMF: ^#^ *p* < 0.05.

### 3.3 Plantarflexor muscle function

**FIGURE 4:**
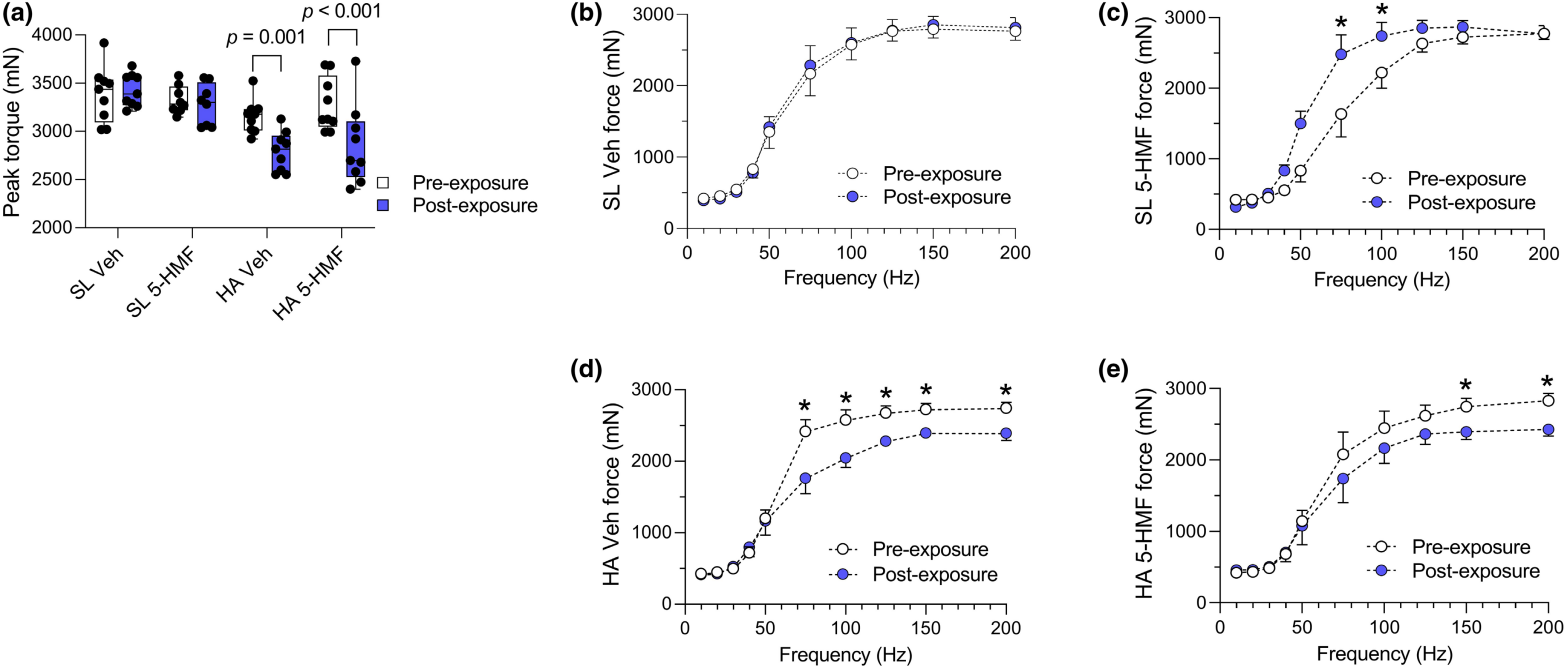
Plantarflexor muscle function measured in anesthetized rats breathing normobaric normoxic or hypoxic gas. (a) Peak torque measured at 175 Hz. Values are individual data points (millinewtons, mN) with interquartile ranges (whiskers represent min to max values), *n* = 8–9/group. Main effect of HA and time (*p* < 0.001), and HA × time (*p* = 0.007). Force‐frequency curves in (a) SL Veh, (b) SL 5‐HMF, (c) HA Veh, and (d) HA 5‐HMF rats. Values are means (mN) ± SE, *n* = 8‐9/group. Main effect of time within groups, *p < 0.05.

Pre‐exposure peak torque measured at 175 Hz was similar between groups (Figure 4a). When measurements were repeated after exposures, HA control and 5‐HMF rats showed similar reductions (−13%) in peak torque. Force‐frequency measurements followed a different pattern (Figure 4b–e). At sea level, 5‐HMF increased mean force production at 75 and 100 Hz by 844 and 519 mN, respectively. After HA exposure, force production decreased in control rats by 14%–37% at frequencies from 75 to 200 Hz, but only at 150 and 200 Hz (−15% to −17%) in 5‐HMF rats.

### 3.4 Superoxide levels and ΔΨm

**FIGURE 5:**
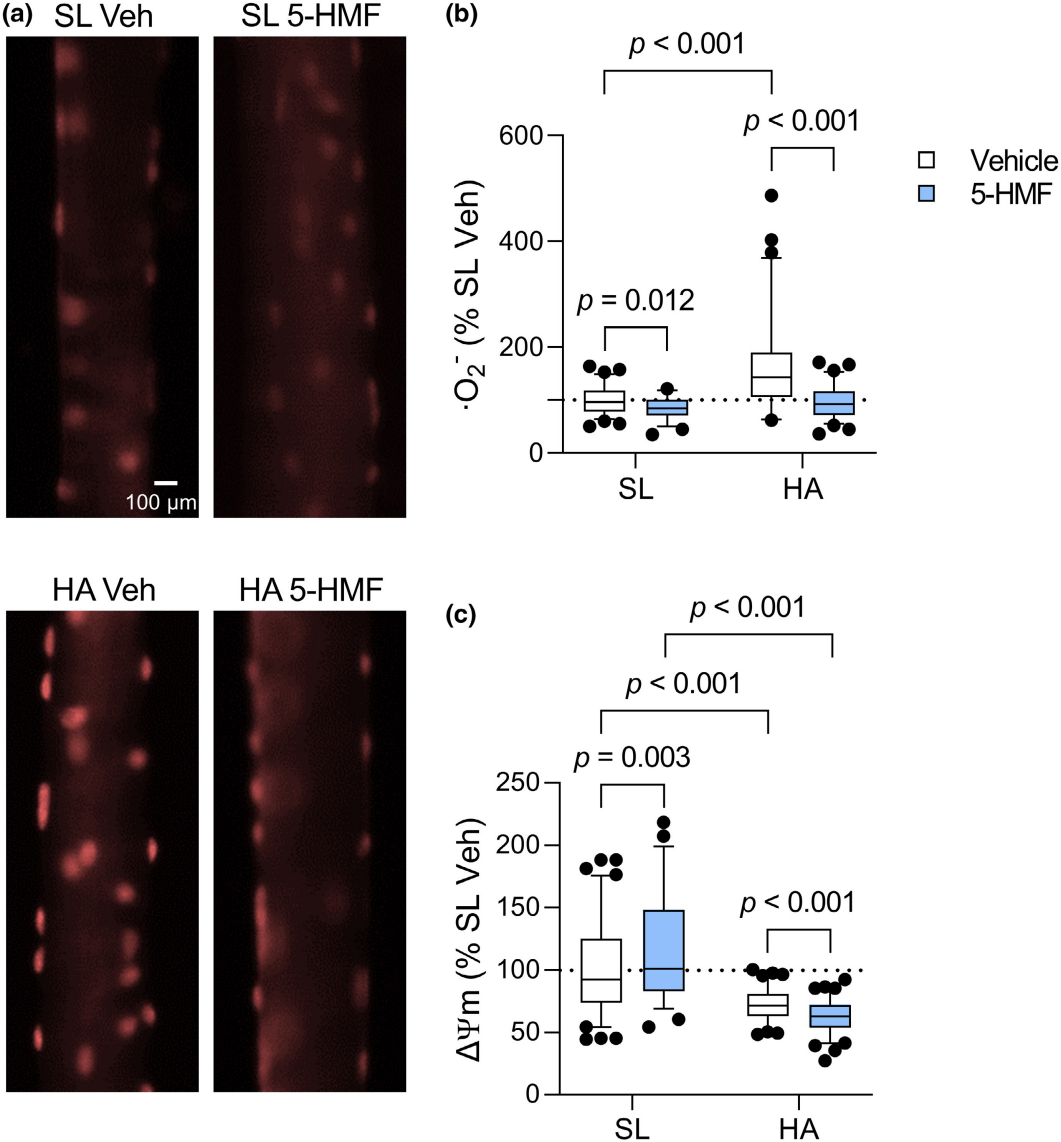
FDB myofiber superoxide (·O_2_^−^) levels and ΔΨm. (a) Representative fluorescent microscopy images of myofibers labeled with DHE. (b) Quantification of DHE fluorescence intensity normalized to SL Veh. (c) Quantification of TMRE fluorescence intensity normalized to SL Veh. Values are interquartile ranges with 5th and 95th percentiles (whiskers), *n* = 50–90 myofibers/group. Main effect of HA, 5‐HMF, and HA × 5‐HMF for DHE, *p* < 0.001. Main effect of HA and HA × 5‐HMF for TMRE, *p* < 0.001.

In myofibers isolated from HA rats, superoxide levels increased by 40% and ΔΨm decreased by 38% compared to sea level controls (Figure 5). 5‐HMF reduced superoxide levels (−15%) and increased ΔΨm (+15%) at sea level, and prevented the increase in superoxide anions while further decreasing ΔΨm in HA rats.

### 3.5 Electron transport chain subunits and mitochondrial fission and fusion regulation

**FIGURE 6:**
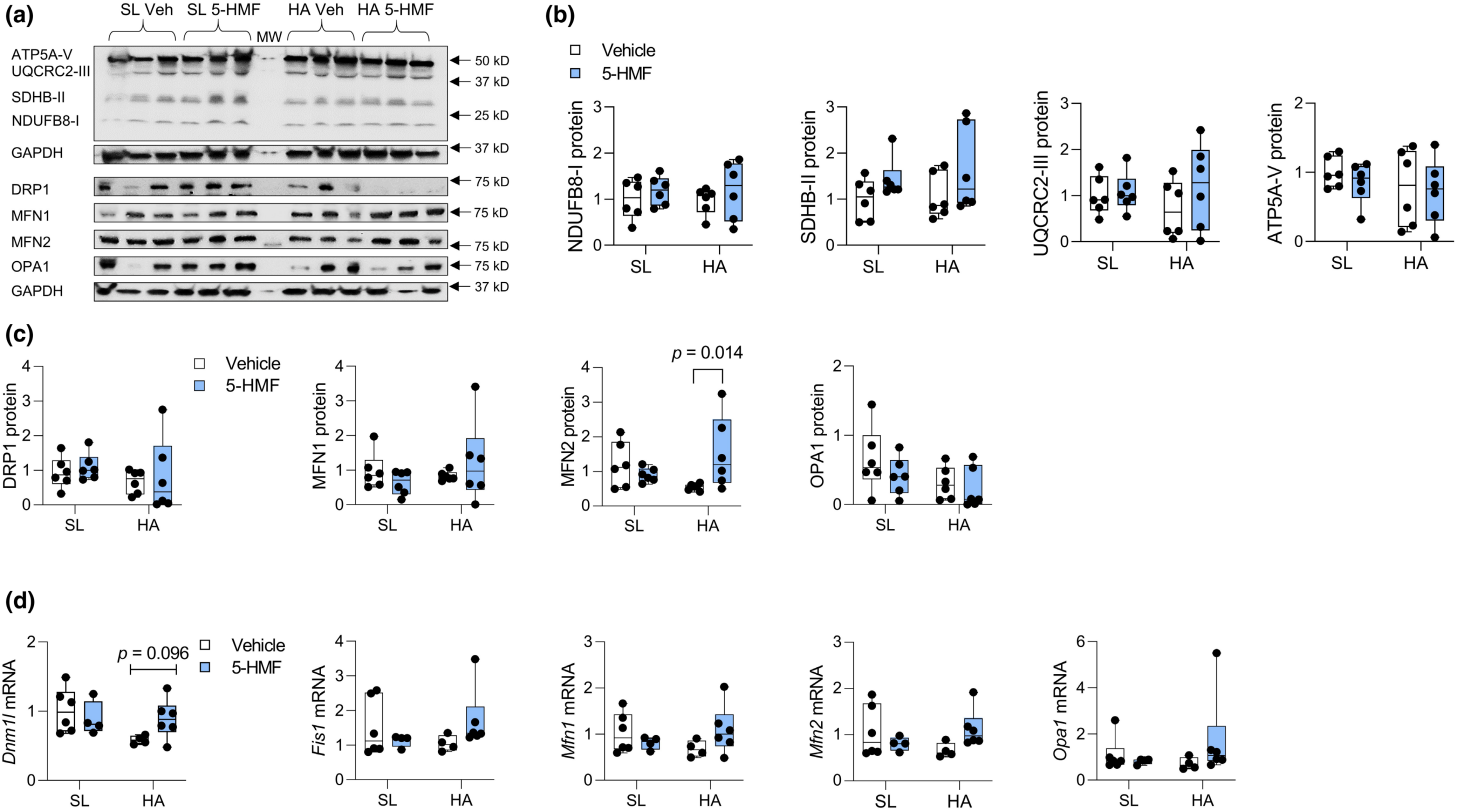
Soleus muscle protein and mRNA expression of mitochondrial complexes and mitochondrial fission and fusion regulators. (a) Densitometry images of complex I subunit I NDUFB8, complex II subunit SDHB, complex III subunit UQCRC2, complex IV subunit ATP5A, DRP1, MFN1, MFN2, OPA1, and GAPDH. Middle lane is MW marker. MW values on right side are marker migration. (b) Quantification of subunits within mitochondrial complexes. (c) Quantification of mitochondrial fission and fusion regulatory proteins normalized to GAPDH. (d) Quantification of mitochondrial fission and fusion regulatory mRNA. Values are individual data points with medians and quartiles (whiskers represent min to max values), *n* = 6/group. Main effect of HA × 5‐HMF for MFN2, *p* = 0.021.

In the soleus muscle, expression of protein subunits within mitochondrial complexes I, II, III, and V, and mitochondrial fission and fusion regulatory proteins were not influenced by HA or 5‐HMF (Figure 6a–c). Of the measured fission and fusion control proteins, MFN2 expression increased by 179% in HA exposed rats treated with 5‐HMF compared to HA controls. There was a trend for a reduction in *Dnm1l*mRNA expression (Figure 6d).

## 4 DISCUSSION

Increasing oxygen binding to hemoglobin in HA should lower mitochondrial oxidant stress and improve mitochondrial activity. 5‐HMF has been shown to increase blood and tissue oxygen levels and reduces oxidant stress, leading us to test this compound in rats exposed to very HA for 72 h. From this work we report the novel finding that 5‐HMF reduced the hypoxic increase in skeletal muscle superoxide levels and prevented decreased plantarflexor isometric force production at some submaximal contraction frequencies (75–125 Hz). Continuous delivery of 5‐HMF, however, did not improve SaO_2_, suggesting its mainly acting as an antioxidant when continuously delivered.

Hypoxia induces a paradoxical increase in oxidants primarily from cytochrome c oxidase, maybe because electron transfer from semiquinone to cytochromes b_L_ and b_H_ is slowed (Burtscher et al., 2022; Guzy et al., 2005; Magalhães et al., 2005). Other sources may include succinate dehydrogenase (complex II), nicotinamide adenine dinucleotide phosphate oxidase, nitric oxide synthase and xanthine oxidase (Burtscher et al., 2022). Here, HA increased superoxide levels in FDB myofibers. Superoxide anions and/or its derivatives, e.g., hydrogen peroxide and hydroxyl radical, increase during isometric muscle contractions and are related to fatigue (Diaz et al., 1993; Kolbeck et al., 1997; Reid et al., 1992). Antioxidants attenuate the reduction in isometric force production during and after repeated muscle contractions (Reid et al., 1992; Shindoh et al., 1990). Thus, it is plausible that oxidants were involved in reducing isometric force production because 5‐HMF prevented the decline at some submaximal contraction frequencies. Moreover, PaO_2_ and SaO_2_, weight loss and atrophy of the plantarflexors, and hemoconcentration were similar between HA groups. 5‐HMF also lowered superoxide levels and augmented force output at submaximal frequencies in sea level rats. These data imply oxidants influence plantarflexor force production during submaximal contractions that is exacerbated in hypoxia.

Reduced peak torque and force output at high frequencies of stimulation in HA exposed rats can be attributed to substantial weight loss and atrophy of the plantarflexor muscles. The primary cause is hypoxic hypophagia that occurs rapidly in rats, and in humans exposed to HA (Aeberli et al., 2013; Schnakenberg & Rogers, 1982). Physical activity was not measured here, but HA exposed rats were lethargic and this may have contributed to HA cachexia. However, this would serve as an adaptive response to conserve energy (Mortola et al., 1994). Muscle atrophy is due to accelerated protein degradation versus changes in protein synthesis rates, resulting in loss of myofibrillar and mitochondrial protein (Chaudhary et al., 2012; Zou et al., 2014, 2015). A certain degree of glycogen and water loss would be expected to accompany proteolysis.

A decrease in mitochondrial content can impact oxidative phosphorylation capacity (Howald et al., 1990; Zou et al., 2015). A fall in ΔΨm during energy restriction limits superoxide leak while meeting ATP demand (López‐Lluch et al., 2006). HA exposure reduced ΔΨm, the main contributor to the electrochemical gradient and ATP production (Brand & Nicholls, 2011), and may have affected maximal torque/force production. Mitochondrial membrane depolarization, however, only controlled superoxide production in 5‐HMF treated animals. Whether the further decline in ΔΨm was a consequence of 5‐HMF's antioxidant activity or a different mechanism cannot be answered here. Accompanying this response was an increase in soleus MFN2, a GTPase that mediates fusion of the outer mitochondrial membrane and preserves the mitochondrial network (Rojo et al., 2002; Wai & Langer, 2016). These findings may imply that in HA 5‐HMF lessens mitochondrial damage by reducing superoxide levels, similarly shown for other antioxidants (vitamin E, dihydromyricetin, and myricetin) (Magalhães et al., 2005;Zou et al., 2014, 2015). Still, the degree of plantarflexor muscle wasting outweighed any effect of 5‐HMF at maximal torque and high frequencies.

In contrast to other studies (Mahon et al., 2019; Yalcin & Cabrales, 2012), 5‐HMF did not raise SaO_2_ at the time of measurement. This may be due the method of delivery. Implanted osmotic pumps provide continuous release of its contents over the duration of the experiment. Based on pre‐exposure body weights and an initial blood volume of 13 mL (Lee & Blaufox, 1985), the rats should have only received 6.25 mg (3.8 mM) of 5‐HMF per min compared to an immediate 9 g (5.5 M) with intravenous infusion. Alternatively, the lack of an effect of 5‐HMF on SaO_2_ may be due to the time and method of blood collection, that is, post‐HA during anesthesia.

### 4.1 Limitations and conclusions

There are limitations for this research that deserve comment. First, in situ plantarflexor muscle function could not be measured during hypobaric hypoxia. We did, however, test the animals at sea level using a similar FiO_2_. Second, arterial blood gas was measured in anesthetized animals maintained on the same normoxic or hypoxic breathing gas. While the values here are similar to previous reports (Jacobson & Dallman, 1989; Wilding et al., 2017), it would have been preferable to perform measurements in conscious rats throughout exposures. This was not technically possible. Third, DHE is a measure of total cellular superoxide and we cannot conclude the source is solely mitochondria. Fourth, mitochondrial dynamic regulatory proteins and mRNA expression were measured in the highly oxidative soleus muscle that did not atrophy to the same extent as the gastrocnemius and plantaris muscles. The lack of a decrease in mitochondrial complex I, II, III, and V protein expression suggests mitochondrial content was maintained in this muscle. A different pattern may exist within the gastrocnemius and plantaris muscles (Zou et al., 2014, 2015). Fifth, statistical power and sample size estimates were based on peak isometric torque, thus we may be underpowered for other measurements. Finally, only males were studied and it may be that female rats respond differently to 5‐HMF at HA.

In conclusion, our data indicate that continuous delivery of 5‐HMF attenuates HA decrements in isometric force production at some submaximal contractions, potentially by limiting superoxide production. Preservation of the mitochondrial network may also be involved. While an effect of 5‐HMF on blood and tissue oxygenation cannot be ruled out, its antioxidant activity demonstrated here warrants further study.

## AUTHOR CONTRIBUTIONS

Geoffrey E. Ciarlone was inolved in was inolved in investigation, data curation, formal analysis, writing–original draft preparation. Joshua M. Swift was inolved in conceptualization, methodology, investigation, writing–review & editing. Brian T. Williams was inolved in investigation, writing–review & editing. Richard T. Mahon was inolved in conceptualization, methodology, investigation, writing–review & editing. Nicholas G. Roney was inolved in conceptualization, methodology, funding acquisition, writing–review & editing. Tianzheng Yu was inolved in methodology, investigation, data curation, writing–review & editing. Heath G. Gasier was inolved in conceptualization, methodology, investigation, formal analysis, writing–original draft preparation.

## FUNDING INFORMATION

This work was supported by the Office of Naval Research #601152 N.0000.000.A1244 (Nicholas G. Roney).

## CONFLICT OF INTEREST STATEMENT

No competing financial interests exist.

## DISCLAIMER

“The views expressed in this journal article are those of the author and do not necessarily reflect the official policy or position of the Department of the Navy, Department of Defense, nor the U.S. Government. The study protocol was reviewed and approved by the Walter Reed Army Institute of Research/Naval Medical Research Center Institutional Animal Care and Use Committee in compliance with all applicable Federal regulations governing the protection of animals in research. The experiments reported herein were conducted in compliance with the Animal Welfare Act and per the principles set forth in the “Guide for Care and Use of Laboratory Animals,” Institute of Laboratory Animals Resources, National Research Council, National Academy Press, 2011. The contents of this publication are the sole responsibility of the author(s) and do not necessarily reflect the views, opinions or policies of Uniformed Services University of the Health Sciences, The Henry M. Jackson Foundation for the Advancement of Military Medicine, Inc., the Department of Defense (DoD), the Departments of the Army, Navy, or Air Force. Mention of trade names, commercial products, or organizations does not imply endorsement by the U.S. Government. Some of the authors are military Service members or employees of the U.S. Government. This work was prepared as part of their official duties. Title 17, U.S.C., §105 provides that copyright protection under this title is not available for any work of the U.S. Government. Title 17, U.S.C., §101 defines a U.S. Government work as a work prepared by a military Service member or employee of the U.S. Government as part of that person's official duties”.

## References

[phy215743-bib-0001] Abdulmalik, O. , Safo, M. K. , Chen, Q. , Yang, J. , Brugnara, C. , Ohene‐Frempong, K. , Abraham, D. J. , & Asakura, T. (2005). 5‐Hydroxymethyl‐2‐furfural modifies intracellular sickle haemoglobin and inhibits sickling of red blood cells. British Journal of Haematology, 128, 552–561. 15686467 10.1111/j.1365-2141.2004.05332.x

[phy215743-bib-0002] Abraham, K. , Gurtler, R. , Berg, K. , Heinemeyer, G. , Lampen, A. , & Appel, K. E. (2011). Toxicology and risk assessment of 5‐Hydroxymethylfurfural in food. Molecular Nutrition & Food Research, 55, 667–678. 21462333 10.1002/mnfr.201000564

[phy215743-bib-0003] Aeberli, I. , Erb, A. , Spliethoff, K. , Meier, D. , Gotze, O. , Fruhauf, H. , Fox, M. , Finlayson, G. S. , Gassmann, M. , Berneis, K. , Maggiorini, M. , Langhans, W. , & Lutz, T. A. (2013). Disturbed eating at high altitude: Influence of food preferences, acute mountain sickness and satiation hormones. European Journal of Nutrition, 52, 625–635. 22573211 10.1007/s00394-012-0366-9

[phy215743-bib-0004] Balsom, P. D. , Gaitanos, G. C. , Ekblom, B. , & Sjodin, B. (1994). Reduced oxygen availability during high intensity intermittent exercise impairs performance. Acta Physiologica Scandinavica, 152, 279–285. 7872005 10.1111/j.1748-1716.1994.tb09807.x

[phy215743-bib-0005] Bloemberg, D. , & Quadrilatero, J. (2012). Rapid determination of myosin heavy chain expression in rat, mouse, and human skeletal muscle using multicolor immunofluorescence analysis. PLoS One, 7, e35273. 22530000 10.1371/journal.pone.0035273 PMC3329435

[phy215743-bib-0006] Bowtell, J. L. , Cooke, K. , Turner, R. , Mileva, K. N. , & Sumners, D. P. (2014). Acute physiological and performance responses to repeated sprints in varying degrees of hypoxia. Journal of Science and Medicine in Sport, 17, 399–403. 23809839 10.1016/j.jsams.2013.05.016

[phy215743-bib-0007] Brand, M. D. , & Nicholls, D. G. (2011). Assessing mitochondrial dysfunction in cells. The Biochemical Journal, 435, 297–312. 21726199 10.1042/BJ20110162 PMC3076726

[phy215743-bib-0008] Burtscher, J. , Mallet, R. T. , Pialoux, V. , Millet, G. P. , & Burtscher, M. (2022). Adaptive responses to hypoxia and/or Hyperoxia in humans. Antioxidants & Redox Signaling, 37, 887–912. 35102747 10.1089/ars.2021.0280

[phy215743-bib-0009] Calbet, J. A. , & Lundby, C. (2009). Air to muscle O_2_ delivery during exercise at altitude. High Altitude Medicine & Biology, 10, 123–134. 19555296 10.1089/ham.2008.1099

[phy215743-bib-0011] Chandel, N. S. , McClintock, D. S. , Feliciano, C. E. , Wood, T. M. , Melendez, J. A. , Rodriguez, A. M. , & Schumacker, P. T. (2000). Reactive oxygen species generated at mitochondrial complex III stabilize hypoxia‐inducible factor‐1alpha during hypoxia: A mechanism of O_2_ sensing. The Journal of Biological Chemistry, 275, 25130–25138. 10833514 10.1074/jbc.M001914200

[phy215743-bib-0012] Chaudhary, P. , Suryakumar, G. , Prasad, R. , Singh, S. N. , Ali, S. , & Ilavazhagan, G. (2012). Chronic hypobaric hypoxia mediated skeletal muscle atrophy: Role of ubiquitin‐proteasome pathway and calpains. Molecular and Cellular Biochemistry, 364, 101–113. 22215202 10.1007/s11010-011-1210-x

[phy215743-bib-0013] Diaz, P. T. , She, Z. W. , Davis, W. B. , & Clanton, T. L. (1993). Hydroxylation of salicylate by the in vitro diaphragm: Evidence for hydroxyl radical production during fatigue. Journal of Applied Physiology, 1985(75), 540–545. 10.1152/jappl.1993.75.2.540 8226451

[phy215743-bib-0015] Dohl, J. , Foldi, J. , Heller, J. , Gasier, H. G. , Deuster, P. A. , & Yu, T. (2018). Acclimation of C2C12 myoblasts to physiological glucose concentrations for in vitro diabetes research. Life Sciences, 211, 238–244. 30253137 10.1016/j.lfs.2018.09.041

[phy215743-bib-0017] Gandra, P. G. , Shiah, A. A. , Nogueira, L. , & Hogan, M. C. (2018). A mitochondrial‐targeted antioxidant improves myofilament Ca(2^+^) sensitivity during prolonged low frequency force depression at low PO_2_ . The Journal of Physiology, 596, 1079–1089. 29334129 10.1113/JP275470 PMC5851896

[phy215743-bib-0018] Girard, O. , Brocherie, F. , & Millet, G. P. (2016). High altitude increases alteration in maximal torque but not in rapid torque development in knee extensors after repeated treadmill sprinting. Frontiers in Physiology, 7, 97. 27014095 10.3389/fphys.2016.00097 PMC4789550

[phy215743-bib-0019] Goods, P. S. , Dawson, B. T. , Landers, G. J. , Gore, C. J. , & Peeling, P. (2014). Effect of different simulated altitudes on repeat‐sprint performance in team‐sport athletes. International Journal of Sports Physiology and Performance, 9, 857–862. 24509626 10.1123/ijspp.2013-0423

[phy215743-bib-0020] Guzy, R. D. , Hoyos, B. , Robin, E. , Chen, H. , Liu, L. , Mansfield, K. D. , Simon, M. C. , Hammerling, U. , & Schumacker, P. T. (2005). Mitochondrial complex III is required for hypoxia‐induced ROS production and cellular oxygen sensing. Cell Metabolism, 1, 401–408. 16054089 10.1016/j.cmet.2005.05.001

[phy215743-bib-0022] Hogan, M. C. , Richardson, R. S. , & Haseler, L. J. (1999). Human muscle performance and PCr hydrolysis with varied inspired oxygen fractions: A 31P‐MRS study. Journal of Applied Physiology, 1985(86), 1367–1373. 10.1152/jappl.1999.86.4.1367 10194224

[phy215743-bib-0023] Howald, H. , Pette, D. , Simoneau, J. A. , Uber, A. , Hoppeler, H. , & Cerretelli, P. (1990). Effect of chronic hypoxia on muscle enzyme activities. International Journal of Sports Medicine, 11(Suppl 1), S10–S14. 2323857 10.1055/s-2007-1024847

[phy215743-bib-0024] Jacobson, L. , & Dallman, M. F. (1989). ACTH secretion and ventilation increase at similar arterial PO_2_ in conscious rats. Journal of Applied Physiology, 1985(66), 2245–2250. 10.1152/jappl.1989.66.5.2245 2545658

[phy215743-bib-0026] Kolbeck, R. C. , She, Z. W. , Callahan, L. A. , & Nosek, T. M. (1997). Increased superoxide production during fatigue in the perfused rat diaphragm. American Journal of Respiratory and Critical Care Medicine, 156, 140–145. 9230738 10.1164/ajrccm.156.1.9610041

[phy215743-bib-0027] Komiya, Y. , Anderson, J. E. , Akahoshi, M. , Nakamura, M. , Tatsumi, R. , Ikeuchi, Y. , & Mizunoya, W. (2015). Protocol for rat single muscle fiber isolation and culture. Analytical Biochemistry, 482, 22–24. 25912416 10.1016/j.ab.2015.03.034

[phy215743-bib-0028] Lee, H. B. , & Blaufox, M. D. (1985). Blood volume in the rat. Journal of Nuclear Medicine, 26, 72–76. 3965655

[phy215743-bib-0029] Li, M. M. , Wu, L. Y. , Zhao, T. , Wu, K. W. , Xiong, L. , Zhu, L. L. , & Fan, M. (2011). The protective role of 5‐hydroxymethyl‐2‐furfural (5‐HMF) against acute hypobaric hypoxia. Cell Stress & Chaperones, 16, 529–537. 21494793 10.1007/s12192-011-0264-8 PMC3156263

[phy215743-bib-0030] López‐Lluch, G. , Hunt, N. , Jones, B. , Zhu, M. , Jamieson, H. , Hilmer, S. , Cascajo, M. , Allard, J. , Ingram, D. K. , & Navas, P. (2006). Calorie restriction induces mitochondrial biogenesis and bioenergetic efficiency. Proceedings of the National Academy of Sciences, 103, 1768–1773. 10.1073/pnas.0510452103 PMC1413655 16446459

[phy215743-bib-0032] Magalhães, J. , Ascensão, A. , Soares, J. M. , Ferreira, R. , Neuparth, M. J. , Marques, F. , & Duarte, J. A. (2005). Acute and severe hypobaric hypoxia increases oxidative stress and impairs mitochondrial function in mouse skeletal muscle. Journal of Applied Physiology, 99, 1247–1253. 15905323 10.1152/japplphysiol.01324.2004

[phy215743-bib-0033] Mahon, R. T. , Ciarlone, G. E. , Roney, N. G. , & Swift, J. M. (2019). Cardiovascular parameters in a swine model of normobaric hypoxia treated with 5‐Hydroxymethyl‐2‐furfural (5‐HMF). Frontiers in Physiology, 10, 395. 31057414 10.3389/fphys.2019.00395 PMC6482156

[phy215743-bib-0034] Morales‐Alamo, D. , Losa‐Reyna, J. , Torres‐Peralta, R. , Martin‐Rincon, M. , Perez‐Valera, M. , Curtelin, D. , Ponce‐Gonzalez, J. G. , Santana, A. , & Calbet, J. A. (2015). What limits performance during whole‐body incremental exercise to exhaustion in humans? The Journal of Physiology, 593, 4631–4648. 26250346 10.1113/JP270487 PMC4606539

[phy215743-bib-0035] Mortola, J. P. , Matsuoka, T. , Saiki, C. , & Naso, L. (1994). Metabolism and ventilation in hypoxic rats: Effect of body mass. Respiration Physiology, 97, 225–234. 7938919 10.1016/0034-5687(94)90028-0

[phy215743-bib-0036] Murphy, M. P. (2009). How mitochondria produce reactive oxygen species. Biochemical Journal, 417, 1–13. 19061483 10.1042/BJ20081386 PMC2605959

[phy215743-bib-0037] Ou, L. C. , Sardella, G. L. , Hill, N. S. , & Tenney, S. M. (1986). Acute and chronic pulmonary pressor responses to hypoxia: The role of blunting in acclimatization. Respiration Physiology, 64, 81–91. 3704382 10.1016/0034-5687(86)90062-9

[phy215743-bib-0038] Parolin, M. L. , Spriet, L. L. , Hultman, E. , Hollidge‐Horvat, M. G. , Jones, N. L. , & Heigenhauser, G. J. (2000). Regulation of glycogen phosphorylase and PDH during exercise in human skeletal muscle during hypoxia. American Journal of Physiology. Endocrinology and Metabolism, 278, E522–E534. 10710508 10.1152/ajpendo.2000.278.3.E522

[phy215743-bib-0039] Reid, M. B. (2016). Reactive oxygen species as agents of fatigue. Medicine and Science in Sports and Exercise, 48, 2239–2246. 27285492 10.1249/MSS.0000000000001006

[phy215743-bib-0040] Reid, M. B. , Haack, K. E. , Franchek, K. M. , Valberg, P. A. , Kobzik, L. , & West, M. S. (1992). Reactive oxygen in skeletal muscle. I. Intracellular oxidant kinetics and fatigue in vitro. Journal of Applied Physiology, 73, 1797–1804. 1474054 10.1152/jappl.1992.73.5.1797

[phy215743-bib-0041] Richardson, R. S. , Duteil, S. , Wary, C. , Wray, D. W. , Hoff, J. , & Carlier, P. G. (2006). Human skeletal muscle intracellular oxygenation: The impact of ambient oxygen availability. The Journal of Physiology, 571, 415–424. 16396926 10.1113/jphysiol.2005.102327 PMC1796788

[phy215743-bib-0042] Rojo, M. , Legros, F. , Chateau, D. , & Lombes, A. (2002). Membrane topology and mitochondrial targeting of mitofusins, ubiquitous mammalian homologs of the transmembrane GTPase Fzo. Journal of Cell Science, 115, 1663–1674. 11950885 10.1242/jcs.115.8.1663

[phy215743-bib-0043] Schnakenberg, D. D. , & Rogers, Q. R. (1982). Effects of time and duration of exposure to 12% O2 and prior food deprivation on hypoxic hypophagia of rats. Aviation, Space, and Environmental Medicine, 53, 1202–1206. 7159341

[phy215743-bib-0044] Shapla, U. M. , Solayman, M. , Alam, N. , Khalil, M. I. , & Gan, S. H. (2018). 5‐Hydroxymethylfurfural (HMF) levels in honey and other food products: Effects on bees and human health. Chemistry Central Journal, 12, 35. 29619623 10.1186/s13065-018-0408-3 PMC5884753

[phy215743-bib-0045] Shindoh, C. , DiMarco, A. , Thomas, A. , Manubay, P. , & Supinski, G. (1990). Effect of N‐acetylcysteine on diaphragm fatigue. Journal of Applied Physiology, 1985(68), 2107–2113. 10.1152/jappl.1990.68.5.2107 2361912

[phy215743-bib-0046] Swift, J. M. , Nilsson, M. I. , Hogan, H. A. , Sumner, L. R. , & Bloomfield, S. A. (2010). Simulated resistance training during hindlimb unloading abolishes disuse bone loss and maintains muscle strength. Journal of Bone and Mineral Research, 25, 564–574. 19653816 10.1359/jbmr.090811

[phy215743-bib-0049] Wai, T. , & Langer, T. (2016). Mitochondrial dynamics and metabolic regulation. Trends in Endocrinology and Metabolism, 27, 105–117. 26754340 10.1016/j.tem.2015.12.001

[phy215743-bib-0050] Wilding, L. A. , Hampel, J. A. , Khoury, B. M. , Kang, S. , Machado‐Aranda, D. , Raghavendran, K. , & Nemzek, J. A. (2017). Benefits of 21% oxygen compared with 100% oxygen for delivery of isoflurane to mice (*Mus musculus*) and rats (*Rattus norvegicus*). Journal of the American Association for Laboratory Animal Science, 56, 148–154. 28315643 PMC5361039

[phy215743-bib-0051] Woyke, S. , Mair, N. , Ortner, A. , Haller, T. , Ronzani, M. , Rugg, C. , Strohle, M. , Wintersteiger, R. , & Gatterer, H. (2021). Dose‐ and sex‐dependent changes in hemoglobin oxygen affinity by the micronutrient 5‐hydroxymethylfurfural and alpha‐ketoglutaric acid. Nutrients, 13(10), 3448. 34684449 10.3390/nu13103448 PMC8537252

[phy215743-bib-0052] Yalcin, O. , & Cabrales, P. (2012). Increased hemoglobin O_2_ affinity protects during acute hypoxia. American Journal of Physiology‐Heart and Circulatory Physiology, 303, H271–H281. 22636677 10.1152/ajpheart.00078.2012 PMC3423161

[phy215743-bib-0053] Yu, T. , Dohl, J. , Chen, Y. , Gasier, H. G. , & Deuster, P. A. (2019). Astaxanthin but not quercetin preserves mitochondrial integrity and function, ameliorates oxidative stress, and reduces heat‐induced skeletal muscle injury. Journal of Cellular Physiology, 234, 13292–13302. 30609021 10.1002/jcp.28006 PMC13483336

[phy215743-bib-0054] Zou, D. , Chen, K. , Liu, P. , Chang, H. , Zhu, J. , & Mi, M. (2014). Dihydromyricetin improves physical performance under simulated high altitude. Medicine and Science in Sports and Exercise, 46, 2077–2084. 24637344 10.1249/MSS.0000000000000336

[phy215743-bib-0055] Zou, D. , Liu, P. , Chen, K. , Xie, Q. , Liang, X. , Bai, Q. , Zhou, Q. , Liu, K. , Zhang, T. , Zhu, J. , & Mi, M. (2015). Protective effects of myricetin on acute hypoxia‐induced exercise intolerance and mitochondrial impairments in rats. PLoS One, 10, e0124727. 25919288 10.1371/journal.pone.0124727 PMC4412664

